# Impaired Executive Functioning Associated with Alcohol-Related Neurocognitive Disorder including Korsakoff’s Syndrome

**DOI:** 10.3390/jcm12206477

**Published:** 2023-10-12

**Authors:** Gwenny T. L. Janssen, Jos I. M. Egger, Roy P. C. Kessels

**Affiliations:** 1 Centre of Excellence for Korsakoff and Alcohol-Related Cognitive Disorders, Vincent van Gogh Institute for Psychiatry, 5803 DN Venray, The Netherlands; jos.egger@donders.ru.nl (J.I.M.E.); roy.kessels@donders.ru.nl (R.P.C.K.); 2 Centre of Excellence for Neuropsychiatry, Vincent van Gogh Institute for Psychiatry, 5803 DN Venray, The Netherlands; 3 Donders Institute for Brain, Cognition and Behaviour, Radboud University, 6525 GD Nijmegen, The Netherlands; 4 Tactus Addiction Care, 7418 ET Deventer, The Netherlands

**Keywords:** executive functioning, alcohol-induced neurocognitive disorder, Korsakoff’s syndrome, Cambridge Neuropsychological Test Automated Battery, personalized treatment

## Abstract

(1) Background: chronic alcohol use is consistently associated with impaired executive functioning, but its profile across the spectrum from mild to major alcohol-related cognitive impairment is, to date, unclear. This study aims to compare executive performances of patients with alcohol-induced neurocognitive disorder, including Korsakoff’s syndrome (KS), by using a computerized assessment battery allowing a fine-grained and precise neuropsychological assessment; (2) Methods: performances of 22 patients with alcohol-related cognitive impairment (ARCI) and 20 patients with KS were compared to those of 22 matched non-alcoholic controls. All participants were diagnosed in accordance with DSM-5-TR criteria and were at least six weeks abstinent from alcohol prior to assessment. Executive function was evaluated using four subtests of Cambridge Neuropsychological Test Automated Battery (CANTAB^®^); (3) Results: significant differences between groups were found on spatial working memory (updating), sustained attention and inhibitory control, set shifting, and planning. Healthy controls performed significantly better than both patient groups (Games-Howell post hoc; *p* < 0.05), but no differences in performance were found between the ARCI and KS group; (4) Conclusions: ARCI and KS patients showed significant executive impairments, most prominent in updating, set-shifting and general planning abilities. Findings suggest equivalent levels of executive function in ARCI and KS patients. Our results highlight executive function as a significant hallmark of alcohol-induced neurocognitive disorder and stipulate the importance of early assessment and evaluation of skills to guide treatment.

## 1. Introduction

Alcohol Use Disorder (AUD), formerly referred to as alcohol addiction, ranks among the most common mental disorders globally and is characterized by an individual’s impaired capacity to reduce or regulate alcohol consumption despite harmful social, occupational or health consequences [1]. Long-term and excessive alcohol use may potentially lead to brain damage adversely affecting neurocognitive function, referred to as alcohol-induced neurocognitive disorder (APA, 2022). These impairments may range from mild to severe, persist beyond the usual duration of intoxication and acute withdrawal and typically involve a combination of difficulties in the domains of episodic memory, learning, visuospatial abilities, executive function and social cognition [2].

Prevalence estimates of neurocognitive deficits in treatment-seeking patients with AUD vary between 50% and 80% [3]. A small yet significant proportion of individuals with AUD may develop severe and irreversible neurocognitive deficits, typically present in Korsakoff’s syndrome (KS). KS is a disorder of the central nervous system which is caused by vitamin B1 deficiency (thiamine) and usually exists within the context of excessive and prolonged alcohol misuse and malnutrition. KS is characterized by disproportional memory impairments (amnesia affecting encoding, consolidation and retrieval of information) and confabulations, lack of insight, executive dysfunction and personality changes [4,5], while crystallized intellectual functioning (i.e., over-learned abilities and culture-specific knowledge) is relatively spared [6]. 

The cognitive deficits in most AUD patients seeking treatment do not meet the criteria of KS. They may, however, include mild to moderate impairments on tests that measure specific neurocognitive functions. These can be classified as alcohol-related cognitive impairment (ARCI), that is, cognitive impairments that occur in the context of AUD, but have a different cognitive profile than KS (i.e., deficits that are most profound in non-memory cognitive domains and/or memory impairments that are less severe and the result of executive dysfunction rather than amnesia). In a review of the literature, Bates and colleagues [7] identified memory function and fluid abilities, such as concept formation, abstract reasoning and problem solving, to be among the most vulnerable cognitive functions in patients with ARCI. Sachdeva and colleagues [8] showed that in comparison with healthy controls, patients with ARCI performed poorly on measures of visuospatial function, verbal abstract reasoning, letter fluency, working memory and motor speed. Additionally, Le Berre and colleagues [9] argued that treatment-seeking patients with ARCI have memory and executive function impairments, as well as social and emotional processing difficulties. Although evidence suggests partial recovery of cognitive deficits after prolonged cessation of drinking (moderated by factors like age, sex, chronicity, nutritional status and genetics), the rate of recovery varies greatly, and cognitive deficits may persist in some patients even after long-term abstinence [10,11,12]. 

ARCI is thus a heterogeneous concept but appears to especially affect executive function (EF). Executive skills are typically involved in everyday-life tasks such as setting realistic goals, initiating and organizing behaviour, executing plans and in evaluating strategies and reflecting upon outcomes [13]. EF encompasses core cognitive skills, including the updating of working memory representations, shifting between tasks or mental sets and inhibition of dominant or prepotent responses [14,15]. In general, persistent executive impairments may contribute to poor job performance, and can interfere with academic achievement, lifestyle choices and treatment options. For instance, executive dysfunction interferes with motivational processes to abandon excessive drinking patterns, may comprise an individual’s effort towards sustained abstinence, and may increase the risk of relapse. As a result, it may generally impair the ability to benefit from treatment [16,17]. Despite accumulating evidence on the impact of alcohol-related executive disfunction, literature on treatment and treatment outcome is sparse and inconclusive [18]. Increasing our understanding of executive dysfunction in AUD with impairments ranging from severe to subclinical or mild symptoms will provide us with an opportunity to adjust interventions and consider personalized treatment options. The assessment of executive symptoms early in the course of alcohol-induced neurocognitive disorder may be critical for guiding clinicians towards accurate diagnoses and optimal care. 

While executive deficits are often reported in alcoholic KS patients [19,20,21], it is still debated whether executive dysfunction should be considered a ‘core’ function of KS or whether it is due to the alcoholic prefrontal encephalopathy that is also present in patients in the subacute phase of KS—see, e.g., [22,23]. Studies on EF to date have either compared KS patients to healthy controls or ‘uncomplicated’ alcoholics, or compared ARCI patients to healthy controls. Studies that directly compare patients with KS to individuals with ARCI who do not meet the criteria for KS and healthy controls are lacking.

In sum, impaired executive functioning is often reported in alcohol use disorder including Korsakoff’s syndrome. Executive control is essential for our ability to live independently. Deficits may persist after long-term abstinence from alcohol, affecting a patient’s capacity to adapt to changing circumstances, overseeing behavioural consequences and, as a result, avoid full recovery from alcohol addiction [21,24]. Direct comparisons of individuals with KS, ARCI and healthy controls are, to date, lacking. Clarifying the complex relation between executive impairment and chronic alcohol misuse and identifying vulnerable individuals early in the course of alcohol-induced neurocognitive disorder may provide them with the opportunity to tailor interventions to improve AUD treatment outcomes. In this study, we aim to directly compare executive performances of KS, ARCI (not meeting the criteria of KS) and a sample of healthy controls without substance use disorder. We used a computerized neuropsychological assessment battery in order to explore the main subcomponents of EF, including inhibitory control, set shifting, updating and planning. The utility of computer-based assessment batteries is well-established and may optimize clinical trials by reducing measurement error and between test variability [25]. 

## 2. Materials and Methods

### 2.1. Patients

Performances of 22 patients with mild alcohol-related cognitive impairment (ARCI) (age: M = 57.2, SD = 7.4) and 20 patients with alcoholic Korsakoff’s syndrome (age: M = 55.3, SD = 8.9) were compared to those of 22 matched non-alcoholic controls (CON) (age: M = 48.9, SD = 15.7). Patients were recruited from the Centre of Excellence for Korsakoff and Alcohol-Related Cognitive Disorders. The ARCI patients met the DSM-5-TR criteria for mild alcohol-induced neurocognitive disorder (APA, 2022). The KS patients met the criteria for major alcohol-induced neurocognitive disorder, amnestic-confabulatory type (APA, 2022) and the criteria outlined in Kopelman [26]. Diagnoses were substantiated by a thorough review of the patients’ medical history including their nutritional history and a history of Wernicke’s encephalopathy, extensive neuropsychological assessment, neurological examination, neuroradiological evidence and clinical observations. All patients received adequate thiamine replacement and were at least six weeks abstinent from alcohol prior to assessment. All patients were able to communicate in the Dutch language.

Healthy controls were volunteers who participated in research, recruited inside and outside the mental health care services, including researchers’ relatives, volunteers and residential staff members. Exclusion criteria for all healthy participants were a history of substance use disorder, neurological disease or psychiatric disorders, use of psychopharmacological medication, or an inability to communicate in the Dutch language. Education level was recorded using 7 levels in accordance with the Dutch educational system [27]. Intelligence was estimated using the Dutch version of the National Adult Reading Test (NART), a reading test that has been validated as an estimate of crystallized intelligence quotient [28], and long-term memory was assessed using the Dutch version of the Rey Auditory Verbal Learning Test (RAVLT; patient groups only) [29]. Groups did not significantly differ on age or estimated premorbid intelligence scores (*p* ≥ 0.05), but the healthy controls had a slightly higher mean education level. The number of men and woman were evenly distributed across groups (*p* = 0.172). Patients with KS performed significantly worse on both immediate recall and delayed recall of the RAVLT, compared to patients with ARCI (*p* = 0.014 and *p* < 0.001, respectively). Demographic characteristics are shown in Table 1.

### 2.2. Materials

The three main subdomains of executive functioning (shifting, updating and inhibition) were assessed using four subtasks of the Cambridge Neuropsychological Test Automated Battery (CANTAB^®^) [30,31]; that is, Spatial Working Memory (SWM), Intra-Extra Dimensional Set Shift (IED), Rapid Visual Information Processing (RVP) and Stockings of Cambridge (SOC). The CANTAB^®^ is a widely used and validated computer-based cognitive assessment battery and is administered to participants by using a touch screen computer. The subtests used in this study have acceptable reliability coefficients (*r* > 0.070) [32,33,34,35]. Since each subtest has multiple outcome measures, we identified one primary outcome measure for each subtest, but also report the results for the most relevant secondary outcome measures.

In the Spatial Working Memory task (SWM), participants had to search for hidden yellow tokens within a visuospatial layout of boxes of increasing set sizes (max = 12). This task measures working memory updating and also assesses strategy use [36,37,38]. Participants are shown a number of coloured squares (boxes) on the screen. The main outcome measure is the number of between-search errors (i.e., going back to a box that already contained a target in a previous search). The mean time to the first response, the strategy measure (with higher scores reflecting a poorer strategy) [39] and the total number of errors (also including the errors that occur when revisiting an empty box within a search) were the reported secondary outcome measures.

The Intra-Extra Dimensional Set Shift (IED) test is a task that assesses rule acquisition and set shifting (reversal learning), in analogue to tasks such as the Wisconsin Card Sorting Test [40,41]. Participants have to detect which of two presented stimuli on the screen is correct in accordance with an implicit rule using feedback. During the task, the correct stimuli and corresponding rules change. These shifts are initially intra-dimensional (i.e., within a previously relevant dimension) and are later extra-dimensional in nature (i.e., a shift to a dimension that was irrelevant before). The primary outcome measure is the adjusted number of total errors (calculated by adding 25 for each stage not attempted due to failure). In addition, errors made in the intra-dimensional stage (number of errors in blocks 2, 5, 7, 9) and errors made in the extra-dimensional stage (number of errors in blocks 6, 8) were included as secondary measures.

The Rapid Visual information Processing task (RVP) is a task measuring sustained attention and inhibition. A white square is presented in the centre of the screen. Digit sequences (from 2–9) appear in the square ranging. Participants have to identify target sequences as quickly as possible, with increasing difficulty levels. The primary outcome measure is the sensitivity to the target (A’). The secondary outcome measures include the mean latency (speed of response) and the number of false alarms [42].

The Stockings of Cambridge (SOC) is a task based on the Tower of London [43] that measures planning and problem solving and relies on all three core executive functions (updating, inhibition and shifting). Participants have to match a pattern of balls within a ‘stocking’ in as few moves as possible [44,45,46]. The primary outcome measure is the number of problems solved in the minimum number of moves. The secondary outcome measures are the mean initial planning time to solve 2-step and 5-step problems.

### 2.3. Procedure

Data were obtained as part of routine neuropsychological assessment. Written informed consent was obtained in all patients for the re-use of their data for scientific research. In addition, healthy controls were recruited from inside and outside the mental health care services. Prior to assessment, controls received an information letter and signed an informed consent form. This study was approved by the Institutional Review Board of Vincent van Gogh Institute for Psychiatry (CWOP #20092019).

### 2.4. Data Analysis

Analyses were performed using the IBM Statistical Package for the Social Sciences (SPSS) version 25. There were three steps to the data analysis: demographic analysis, assumption checks, and main analysis. Alpha was set at 0.05 throughout. Since nine variables did not meet the normality assumption, ANOVAs and the Kruskal–Wallis test in combination with the Mann–Whitney test were used to analyse the results. When ANOVAs indicated a significant group difference, Game–Howell post-hoc comparisons were performed.

## 3. Results

**Figure 1:**
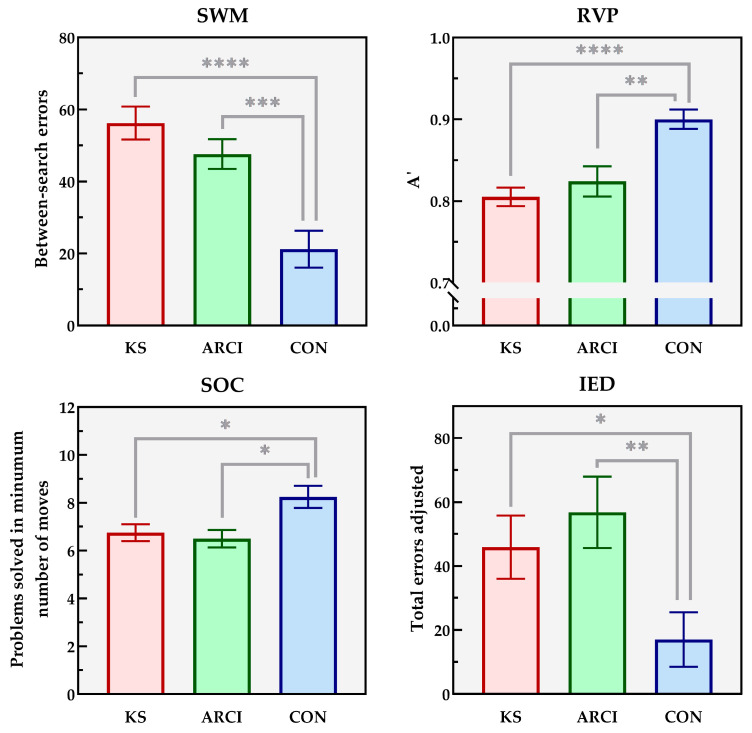
Performance (mean ± SEM) of the patients with Korsakoff syndrome (KS), alcohol-related cognitive impairment (ARCI) and healthy controls (CON) on the primary outcome measures of the Spatial Working Memory (SWM), Rapid Visual Information Processing (RVP), Stockings of Cambridge (SOC) and Intra-Extra Dimensional Set Shift (IED) subtests of the Cambridge Automated Neuropsychological Test Battery (CANTAB^®^). A higher score represents a better performance for the RVP and SOC subtests and a worse performance for the SWM and IED subtests. * *p* < 0.05; ** *p* < 0.01; *** *p* < 0.001; **** *p* < 0.0005.

Figure 1 shows the results of the primary outcome measure for the four executive tests for the KS, ARCI and CON groups. Table 2 displays the secondary outcome measures for each subtest test for the three groups.

In the Spatial Working Memory (SWM) task, significant group differences were found on the number of between-search errors (*F*(2,58) = 15.33; *p* < 0.0005; η*_p_*^2^ = 0.346), total number of errors (*F*(2,58) = 19.90; *p* < 0.0005; η*_p_*^2^ = 0.339), strategy use (*F*(2,57) = 27.60; 82; *p* < 0.0005; η*_p_*^2^ = 0.492) and the mean time to the first response (*F*(2,44) = 13.34; *p* < 0.0005; η*_p_*^2^ = 0.377). The Games–Howell post-hoc analyses showed significantly less between-search errors in the healthy controls compared to both patients with KS (*p* < 0.0005) and patients with ARCI (*p* = 0.001), and significantly less total errors of healthy controls compared to patients with KS (*p* < 0.0005) and patients with ARCI (*p* = 0.001). No significant differences were found between patients with KS and ARCI on the number of between-search errors (*p* = 0.357) or on the number of total errors (*p* = 0.397). Furthermore, healthy controls showed more efficient strategy use compared to patients with KS (*p* = 0.000) and patients with ARCI (*p* < 0.0005), but no differences were found between patients with KS compared to patients with ARCI (*p* = 0.904). Mean time to first response showed significantly faster responses for healthy controls compared to patients with KS (*p* < 0.0005) and patients with ARCI (*p* < 0.0005). Additionally, patients with ARCI responded significantly faster than patient with KS (*p* = 0.030).

On the Rapid Visual Information Processing (RVP) task, a significant group difference was found on the sensitivity to target measure *A*’ (*F*(2,52) = 10.71; *p* < 0.0005; η*_p_*^2^ = 0.292). No significant group differences were found on mean latency scores or on the number of false alarms. The Games–Howell post-hoc analysis showed a significant stronger performance on signal detection (sensitivity to target) of healthy controls compared to patients with KS (*p* < 0.0005) and patients with ARCI (*p* = 0.008). No differences were found between patients with KS and patients with ARCI (*p* = 0.707).

A significant group difference was found on the Stockings of Cambridge (SOC) task for the total number of problems solved in minimal moves (*F*(2,59)= 5.67; *p* = 0.006; η*_p_*^2^ = 0.161), but not on mean initial thinking time for problems solved in a minimum of two moves or for problems solved in a minimum of five moves. The Games–Howell post hoc analysis showed that healthy controls solved significantly more problems in the minimal number of moves compared to patients with KS (*p* = 0.038) and patients with ARCI (*p* = 0.014). No differences were found between patients with KS and patients with ARCI (*p* = 0.876).

Finally, for the Intra-Extra Dimensional Set Shift (IED), significant group differences were found on the number of intra-dimensional errors (*F*(2,58) = 3.36; *p* = 0.047; η*_p_*^2^ = 0.92) and the number of total errors adjusted (*F*(2,50) = 0.001; η*_p_*^2^ = 0.109), but not on the number of extra-dimensional errors. The Games–Howell post hoc analysis indicated a significantly better performance of healthy controls on intra-dimensional set shifting compared to patients with KS (*p* = 0.028), but not compared to patients with ARCI (*p* = 0.335), and significantly less total errors adjusted in the healthy controls compared to patients with KS (*p* = 0.026) and patients with ARCI (*p* = 0.006). No significant differences were found between patients with KS and ARCI on the adjusted number of total errors (*p* = 0.744).

## 4. Discussion

This study aimed to directly compare executive performances in patients with ARCI and KS to a sample of healthy controls using a computerized cognitive assessment battery. Results indicated significantly stronger performances of healthy controls compared to patients with ARCI and KS on both the capacity and efficiency of working memory (updating), inhibitory control (sensitivity to target), set-shifting and overall planning and strategy skills. While healthy controls outperformed the patient groups, no differences were found between the ARCI and KS group on all but one test. Intra-dimensional set-shifting scores showed a significantly stronger performance for controls compared to KS, but not for ARCI. Effect sizes were large for updating (working memory), inhibition and overall planning and strategy skills and moderate for set-shifting skills.

Existing studies have either compared KS patients to healthy controls or ‘uncomplicated’ alcoholics, or compared ARCI patients to healthy controls. Instead, in the current study we examined the performances of KS, ARCI and controls on three subdomains of executive function. Our findings corroborate with previous work on executive deficits in alcohol-induced neurocognitive disorders, including Korsakoff’s syndrome. Harmful effects of chronic alcohol use on executive performances are consistently reported in a substantial proportion of treatment seeking AUD patients—see, e.g., [47,48,49,50,51]. However, as traditional EF tasks are often multi-componential, assessing several subdomains simultaneously, specifying the extent to which specific executive subdomains are involved has proven to be difficult. In an attempt to overcome these methodological issues and to increase the precision of measurement, several authors have adopted a task-based strategy. For example, in a study of Brion and colleagues [24], 47 abstinent alcohol dependent patients were compared to a sample of 47 healthy controls using a nine-task neuropsychological test battery. Participants were matched on age, sex and education level. Findings indicated an overall and significant slowing of speed, vast impairments on shifting and updating skills, while inhibition deficits remained moderate for patient with alcohol dependence. Accordingly, Moerman and colleagues [21] assessed the executive performances of 36 abstinent KS patients and compared these to a sample of 30 healthy controls using tailored computerized EF paradigms. Results showed substantial impairment of shifting and updating skills for KS patients, whereas no significant differences on inhibitory control were found between KS patients and healthy controls. Indeed, our findings indicate that EF deficits exceed the traditionally studied inhibition impairments in alcohol use disorders and included significant difficulties in verbal and visual updating, set-shifting and general planning abilities. In contrast to Moerman et al. [21] and Brion et al. [24], our findings implicated impairments on all levels of executive functioning, including inhibitory control. Differences in task demands may underlie inconsistencies in specific EF profiles across studies, and further exploration is necessary to clarify these discrepancies. Overall, our findings confirm the involvement of the main executive functions in alcohol-induced neurocognitive disorders.

In our sample, KS patients could be clearly differentiated from ARCI patients by their disproportionate memory impairment. However, both patient groups presented with substantial deficits in updating, inhibitory control, general planning skills and, to a lesser extent, set-shifting skills compared to healthy controls. Although KS and ARCI patients showed similar difficulties in the acquisition phase of the set-shifting tasks (i.e., total number of errors adjusted) when compared to controls, KS patients performed significantly worse compared to ARCI and controls on the intra-dimensional shifting phase (reversal learning) where contingencies of rewards are changed. The ability to adjust behaviour in response to changing environmental demands is not only associated with complex goal-directed behaviour, but also with self-awareness, social competence and treatment engagement [52]. In KS, the ability to live an autonomous life is hampered by the often severe cognitive deficits, notably the amnesic syndrome. Executive dysfunction may further complicate independent functioning in daily life. It is worthwhile to follow up on our findings in order to gain more insight into the cumulative bearing of memory impairments and executive deficits, and their relation to the level of independence. This can be done by also assessing everyday memory and executive problems (for instance, using the self-report or informant-based questionnaires such as the Behavior Rating Inventory of Executive Function; BRIEF) [53] as well as observations of everyday function at the level of activities.

Our result also show that EF deficits are not specific for KS, as a similar extent and profile is found in patients with mild to moderate alcohol-induced cognitive disorders. These findings are in line with the neurobiological evidence in patients with alcohol-related cognitive impairments (with or without KS). That is, it has been argued that alcoholic neurotoxicity is associated with frontal lobe pathology, resulting in specific cognitive abilities including executive performances. The thiamine depletion, which is the cause of KS, is associated with a reduced volume of diencephalic and limbic structures, with memory dysfunction as a result [5,54]. As such, executive impairments may appear in all patients with alcohol-induced neurocognitive disorder, including those patients in the subacute phase of alcoholic KS. This notion is in contrast to the continuity hypothesis [55] that postulated a continuous worsening of alcohol-induced cognitive impairments as drinking habits persevere and aggravate. According to the latter, KS patients should perform notably worse on both memory and EF tests when compared to the performances of patient with ARCI. In order to further disentangle the contribution of alcoholic neurotoxicity, psychiatric and physical comorbidity, and thiamine depletion on the pattern of cognitive deficits, future research should systematically examine both non-alcoholic and alcoholic KS patients.

This study is, to our knowledge, the first study to directly compare performances on distinct executive subdomains of patients with KS, ARCI and a sample of healthy controls. We explored executive skills analogue to three factor model of Friedman and Miyake [15] using a computerized test battery. In general, paper and pencil EF tasks are often process impure when assessing several cognitive functions. By selecting computerized tasks targeting specific subdomains of EF analogue to the model as proposed by Miyake [14,15], we were able increase the precision of measurement, cf. [42].

Our study has some limitations that should be addressed. First, our patients were at least six weeks abstinent from alcohol prior to assessment [56]. Nevertheless, it should be noted that evidence on the course and rate of cognitive recovery after (long-term) alcohol abstinence is inconclusive and further recovery after prolonged abstinence is to be expected. While some authors claim full recovery of cognitive functioning in AUD within a year of sobriety, see for example [57], others argue that cognitive impairments can persist well beyond a year of alcohol abstinence. In particular, EF may take longer to recover, with improvements reported up to six years of abstinence [11,12,58]. Second, the current study used a cross-sectional design to identify EF performances in KS, ARCI and controls. Given the scope of the design, limited conclusions can be drawn. Although groups were matched on age, sex and estimated premorbid IQ, assessing group-level EF performances cannot not fully excluding pre-existing (executive) differences. For instance, the control participants had on average a higher education level than the two patient groups. In order to overcome aforementioned limitations, future research should include longitudinal studies employing repeated measures over time and control for potentially confounding factors such as abstinence duration. Finally, although the current patient sample was carefully selected by a thorough review of the patients’ medical history, extensive neuropsychological assessment, neurological examination, neuroradiological evidence and clinical observations, comorbid somatic and mental health issues were not controlled for. It is unclear to what extend these factors could have influenced our results. Future research should include potentially confounding factors such as psychopathological and physical comorbidities.

With respect to the clinical implications of our findings, we argue that assessment of EF is relevant for treatment and care planning. That is, deficits in EF may complicate the addiction treatment of patients with AUD, as they may experience information overload in more complex daily situations, which include group therapy sessions. Adjusting the therapy, for instance, by offering one-on-one sessions or small-group meetings, may thus be required. In patients with more severe alcohol-related cognitive dysfunction, such as patients with KS who are also amnesic, executive deficits may reduce the compensatory strategies that patients with memory deficits without executive dysfunction are still able to employ (such as using a calendar or smartphone to set reminders). This further hampers instrumental activities of daily living, making more structured cognitive rehabilitation approaches more suitable for these patients [59].

## 5. Conclusions

Impaired executive functioning is often reported in individuals with alcohol use disorder, both in mild alcohol-related cognitive impairment and in severely amnesic patients due to Korsakoff’s syndrome. Executive control is essential for our ability to live independently, for motivating alcohol-use cessation, and for maintaining long-term abstinence. Moreover, executive deficits may persist even after long-term abstinence from alcohol. Therefore, assessment of EF over the course of AUD and during abstinence has clinical relevance. This study was the first to directly compare individuals with KS, ARCI, with a sample of healthy controls. Results showed significant EF impairments for individuals with KS and ARCI compared to the healthy controls. Deficits were most prominent in updating, inhibition and general planning abilities. Although KS patients could be differentiated from ARCI patients by their severe memory impairments, no substantial differences between KS and ARCI were found on any of the subdomains of EF. To date, studies that investigated the course of EF in AUD patients that are abstinent for longer periods of time are lacking. Future research on EF in patients with AUD who are at risk of developing ARCI should employ longitudinal designs in order to gain insight into the trajectories of recovery during alcohol abstinence. Furthermore, studies in non-alcoholic KS patients may shed further light on the underlying neurocognitive mechanisms of deficits in EF.

## Figures and Tables

**Table 1:** Demographic variables, intelligence and memory scores for the Korsakoff patients (KS), the alcohol-related cognitive impairment (ARCI) group and the healthy controls (CON).

	KS (*n* = 20)	ARCI (*n* = 22)	CON (*n* = 22)	*p*-Value
Age (mean + SD)	55.3 (8.9)	57.2 (7.4)	48.9 (15.7)	0.050
Sex (% men)	14 (70%)	15 (68.2%)	9 (40.1%)	0.172
Education level (1–7)	3.7 (0.92) ^3^	3.75 (0.86) ^4^	5.59 (0.96)	0.001
NART ^1^-IQ (mean + SD)	92.1 (16.9)	94.5 (19.0)	98.2 (12.5)	0.473
RAVLT T-scores ^2^ (mean + SD)				
Immediate recall	28.4 (11.9)	40.3 (16.2)	n.a.	0.014
Delayed recall	22.1 (7.8)	38.5 (17.4)	n.a.	<0.001

^1^ NART = National Adult Reading Task—Dutch version; ^2^ RAVLT = Rey-Auditory Verbal Learning Test—Dutch version; ^3^ missing in 3 cases; ^4^ missing in 6 cases; n.a. = not available.

**Table 2:** Secondary outcome measures (mean with SD between parentheses) for the Spatial Working Memory (SWM), Rapid Visual Information Processing (RVP), Stockings of Cambridge (SOC) and Intra-Extra Dimensional Set Shift (IED) subtests of the Cambridge Automated Neuropsychological Test Battery (CANTAB^®^) in the patients with Korsakoff syndrome (KS), alcohol-related cognitive impairment (ARCI) and healthy controls (CON).

Task	Outcome Measure	KS	ARCI	CON	Overall *p*-Value	Post-Hoc Comparisons
SWM		*n* = 19	*n* = 22	*n* = 20		
	Strategy measure	37.9 (3.6)	37.1 (8.9)	17.9 (13.6)	< 0.0005	CON > ARCI = KS
	Mean time to first response (ms)	3481 (511)	2446 (683)	1200 (318)	< 0.0005	CON > ARCI > KS
	Total error	57.1 (20.1)	48.7 (20.1)	21.7 (23.7)	< 0.0005	CON > ARCI = KS
RVP		*n* = 18	*n* = 22	*n* = 20		
	Mean latency (ms)	540 (99)	531 (111)	523 (128)	0.901	-
	Number of false alarms	5.78 (8.6)	15.1 (29.9)	2.55 (3.1)	0.074	-
SOC		*n =* 20	*n* = 22	*n* = 20		
	Mean planning time 2-step problems (ms)	2233 (2482)	1463 (1220)	1759 (1788)	0.426	-
	Mean planning time 5-step problems (ms)	3970 (2573)	3154 (1460)	8912 (4894)	0.507	-
IED		*n =* 20	*n* = 22	*n* = 20		
	Intra-dimensional set shifting (blocks 2, 5, 7, 9)	12.1 (9.5)	9.1 (7.4)	6.4 (4.2)	0.047	ARCI = CON; CON > KS
	Extra-dimensional set shifting (blocks 6, 8)	9.5 (9.3)	13.7 (11.0)	11.8 (10.0)	0.402	-

Note. Higher scores represent a worse performance for all secondary outcome measures, except for the SWM strategy score (higher scores reflecting a more efficient strategy).

## Data Availability

Data are available from the corresponding author upon request.
